# SILVER TSUNAMIS, GRAY TIDES, AND DEAD WOOD: EXPLORING AGEIST METAPHORS AT THE INTERSECTION OF AGING AND CLIMATE CHANGE

**DOI:** 10.1093/geroni/igae098.2471

**Published:** 2024-12-31

**Authors:** Ulla Kriebernegg, Anna-Christina Kainradl

**Affiliations:** University of Graz, Graz, Steiermark, Austria; University of Graz, Graz, Steiermark, Austria

## Abstract

This paper explores the ageist metaphorical language that links demographic change and climate change from a cultural gerontological perspective. Older adults are often labeled as ‘vulnerable’ and ‘at risk’ in the face of the climate crisis, framing it as a costly public health challenge. Simultaneously, they face criticism and animosity, being perceived as contributors to environmental degradation. The metaphorical realms of aging and climate change converge in ageist metaphors such as ‘the silver tsunami’ or ‘the gray flood’ that reinforce the image of the double burden allegedly represented by older people. Canadian sociologist Stephen Katz labeled this perspective “alarmist demography,” challenging the notion that the threat arises from a growing, demanding, and relatively affluent group relying on the welfare state, burdening younger generations (1992, 203). Scrutinizing the rising ageism in climate change discourse, this paper illustrates how ageist metaphors might act as a veil, concealing other socio-political issues that warrant attention, such as social inequalities. To showcase this phenomenon, we will use examples from media and film as well as Margaret Atwood’s short story “Torching the Dusties” (2014), which depicts young people setting nursing homes on fire, burning down “the dead wood” to force the old to “make room” for the young. Using such recent cultural representations, we will make the dangerous ageism inherent in the double burden narrative very explicit. To conclude, the paper shows that generational warfare is not helpful, and that tackling climate protection requires a collective effort involving all generations.

